# COVID-19: a new deep learning computer-aided model for classification

**DOI:** 10.7717/peerj-cs.358

**Published:** 2021-02-18

**Authors:** Omar M. Elzeki, Mahmoud Shams, Shahenda Sarhan, Mohamed Abd Elfattah, Aboul Ella Hassanien

**Affiliations:** 1 Faculty of Computers and Information, Mansoura University, Mansoura, Egypt; 2 Faculty of Artificial Intelligence, Kafrelsheikh University, Kafrelsheikh, Egypt; 3 Misr Higher Institute for Commerce and Computers, Mansoura, Egypt; 4 Faculty of Computers and Artificial Intelligence, Cairo University, Egypt, Cairo, Egypt; 5 Scientific Research Group in Egypt (SRGE), Cairo, Egypt

**Keywords:** Deep convolutional neural network, X-ray images, COVID-19, Classification

## Abstract

Chest X-ray (CXR) imaging is one of the most feasible diagnosis modalities for early detection of the infection of COVID-19 viruses, which is classified as a pandemic according to the World Health Organization (WHO) report in December 2019. COVID-19 is a rapid natural mutual virus that belongs to the coronavirus family. CXR scans are one of the vital tools to early detect COVID-19 to monitor further and control its virus spread. Classification of COVID-19 aims to detect whether a subject is infected or not. In this article, a model is proposed for analyzing and evaluating grayscale CXR images called Chest X-Ray COVID Network (CXRVN) based on three different COVID-19 X-Ray datasets. The proposed CXRVN model is a lightweight architecture that depends on a single fully connected layer representing the essential features and thus reducing the total memory usage and processing time verse pre-trained models and others. The CXRVN adopts two optimizers: mini-batch gradient descent and Adam optimizer, and the model has almost the same performance. Besides, CXRVN accepts CXR images in grayscale that are a perfect image representation for CXR and consume less memory storage and processing time. Hence, CXRVN can analyze the CXR image with high accuracy in a few milliseconds. The consequences of the learning process focus on decision making using a scoring function called SoftMax that leads to high rate true-positive classification. The CXRVN model is trained using three different datasets and compared to the pre-trained models: GoogleNet, ResNet and AlexNet, using the fine-tuning and transfer learning technologies for the evaluation process. To verify the effectiveness of the CXRVN model, it was evaluated in terms of the well-known performance measures such as precision, sensitivity, *F*1-score and accuracy. The evaluation results based on sensitivity, precision, recall, accuracy, and F1 score demonstrated that, after GAN augmentation, the accuracy reached 96.7% in experiment 2 (Dataset-2) for two classes and 93.07% in experiment-3 (Dataset-3) for three classes, while the average accuracy of the proposed CXRVN model is 94.5%.

## Introduction

On 31 December 2019, pneumonia of unknown cause found in Wuhan, China, was first confirmed to China’s WHO Country Office, and the disease was named COVID-19 by WHO (World Health Organization, 2020). The WHO declared the Chinese outbreak of COVID-19 on 30 January 2020 to be a public health emergency of international concern posing a high risk to countries with weak healthcare systems. The emergency committee reported that COVID-19 could be stopped by early detection, isolation, timely care, and the implementation of a reliable communication monitoring system (World Health Organization, 2020; Sohrabi et al., 2020). An essential step in combating COVID-19 is the successful monitoring of infected patients, enabling those infected to seek prompt diagnosis and care, as well as being isolated to reduce the spread of the virus. Reverse transcriptase-polymerase chain reaction (RT-PCR) is the principal screening tool used to identify COVID19 cases (Wang, Ng & Brook, 2020).

Smart healthcare systems can assist in improving healthcare worldwide via employing artificial intelligence (AI) and machine learning techniques in different ways, for example, detection, identification, and monitoring of the disease. AI allows doctors to diagnose, discover, and monitor diseases in early stages. In turn, it becomes easier to overcome obstacles and issues in traditional methods such as time consumption and extra effort wasting. From cancer screening and disease tracking to tailored treatment, recommendations are a wide variety of topics and applications in healthcare. These applications depend on different and various sources of today-radiological imaging data (X-ray, CT and MRI scans), pathology imaging, and, more recently, genomic sequences for being used during service(s) implementation (Li et al., 2020; Razzak, Naz & Zaib, 2018; Xu et al., 2014) .

Due to the recent pandemic, the opportunity of smart healthcare expansion is exponentially increasing and attracting many researchers to find a cure or medication to benefit billions of people around the world. Therefore, COVID-19 detection and classification is a critical application in smart healthcare systems (Ting et al., 2020; Ienca & Vayena, 2020; Wang et al., 2020).

In this article, we are motivated to propose an approach that is based on deep learning (DL) technology as a potential tool to assist healthcare workers to be timely to detect the presence of COVID-19 from CXR images.

The main contributions of this article are as follows:

- CXRVN: A novel Chest, X-ray COVID-19 Network architecture, is designed, implemented, trained, optimized, and evaluated to detect infected cases easily, accurately, and rapidly.
- We crawled a novel dataset from different resources, which include COVID-19, Pneumonia, and Normal cases.
- Fine-tuned and transferred learning pre-trained models for feature extraction and image classification.
- Augmentation of the COVID-19 dataset is performed using a designed GANs architecture.

In the rest of this article, we recall the recent scientific researches in literature in “Related Work”. “Materials and Methods” discusses the design principles, learning criteria, and optimization algorithms for our CXRVN model. Validation and verification of the CXRVN model are illustrated using different experimental studies in “Evaluation of Experimental Results”. “Discussion” discusses the results and insights of the experimental studies and compares the performance of CXRVN with recent literature. Finally, “Conclusions” concludes the proposed method and research impacts and highlights additional research questions in the hotspot research point.

## Related Work

COVID-19 has been affecting more than 190 countries and regions since a few months ago. Recently, many attempts by researchers in the field of computer science were introduced and proposed to identify, classify, and diagnose cases, relying on the presence of a limited number of particular databases (Oh, Park & Ye, 2020; Khan, Shah & Bhat, 2020).

Prediction models used to address the pandemic COVID-19 are affected by many different sources, such as the demographics, and issues of vulnerability that can be associated with lung or heart disease, settings/hospital capacity, and the rate of testing, social and spacing, and income in exchange for goods (Santosh, 2020a).

Truncated Inception Network is presented by Das, Santosh & Pal (2020) in order to classify positive CXR images from normal cases, further, they used six different datasets with resulting 99.96% accuracy. The major limitation of this work is there is non-clinical implications performed. They just designed the network to check whether the Truncated Inception Net could be used in detecting COVID-19 positive cases using CXRs. Therefore, recent approaches attempts to use clinical implications for example, nine pregnant women with chest CT-images as limited laboratory-confirmed COVID-19 pneumonia scans were retrospectively reviewed by Chen et al. (2020). Further, The efforts to diagnose and classify each patient and determine whether they had SARS-Co-V-2 infection or not depending upon RT-PCR was presented by Struyf et al. (2020). One more, the study of COVID-19 pneumonia in Wuhan, China, consists of 81 patients who were presented by Shi et al. (2020) depends up on classifying the COVID-19 cases from normal cases.

Different approaches were proposed to address the problem of COVID-19, as well as the variability and concurrent permanent update of corona cases in the databases.

Given the potential future epidemics of COVID-19, AI scientists do not always wait to train complete data sets. Therefore the decision-making process depending not only one data type, but also many data types are used (multimodal data) to ensure the reliability of the AI model to detect the variability of COVID-19 pandemic Santosh (2020b).

In Pereira et al. (2020) proposed a classification scheme based on a multi-class classification and a hierarchical classification where pneumonia can be structured as a hierarchy. Besides, they used resampling algorithms to solve the data imbalance problem during feature extraction by texture descriptors and a pre-trained CNN model from CXR images. They fused the features of two methods to improve the power of several texture descriptors and base classifiers at once. Their hierarchical classifier was tested in RYDLS-20 achieved an *F*1-Score of 0.65 using a multi-class approach and an *F*1-Score of 0.89 for the COVID-19 identification.

Ozturk et al. (2020) presented a new architecture for rapid recognition of COVID-19 using CXR images to provide reliable diagnostic tests for binary classifications (COVID vs. No-Findings) and multi-class classifications (COVID vs. No-Findings vs. Pneumonia). Their model achieved a classification accuracy of 98.08% and 87.02% for binary and multi-class, respectively.

In Ucar & Korkmaz (2020), a new model for the rapid diagnosis of COVID-19 based on deep Bayes-Squeeze Net to overcome the public database imbalance problem, a multi-scale offline increase was performed, and finally an easy-to-install deep learning network for embedded and mobile systems that could assist health experts in establishing a stable system for COVID -19 diagnosis. Their model achieved 98.3% and 100% for multi-class and binary classification, respectively.

Another approach based on deep learning was developed for COVID-19 using the CXR dataset consisting of three classes, namely: normal, COVID-19, and pneumonia was presented by Toğaçar, Ergen & Cömert (2020). Their model starts with a preprocessing step, including restructuring images using the fuzzy color technique. In the next step, the stacked dataset was trained with deep learning models (MobileNetV2 and SqueezeNet), and the feature sets obtained by the models were processed using the Social Mimic optimization method. They trained an SVM using significant features and achieved 99.27% for multi-class classification.

Furthermore, in Apostolopoulos, Aznaouridis & Tzani (2020), MobileNet v2 was used and trained from scratch to investigate the importance of the extracted features of COVID-19. They have been trained CNNs from scratch and improved the other transition learning methods, especially in separating the X-rays using a large-scale dataset of 3905 X-ray images, related to 6 diseases. Their classification model achieved 87.66%, 99.18%, 97.36% and 99.42% for precision, accuracy, sensitivity, and specificity, respectively.

Using statistical analysis of texture feature extraction, Haralick features proposed by Perumal, Narayanan & Rajasekar (2020) are applied to emphasis the region of interest for detecting COVID-19 cases. They used three modalities bacterial pneumonia, viral pneumonia, and normal lung diseases based on transfer learning using VGG16, Resnet50 and Inception V3 and the resulting accuracies are 93.8%, 89.2% and 82.4% respectively.

Fractional-order and marine predators algorithm (FO-MPA) with inception CNN presented by Sahlol et al. (2020) are used to extract the features and classify the COVID-19 chest X-ray (CXR) images respectively. The results obtained are 98.7%, 98.2% and 99.6%, 99% of classification accuracy and F-Score for the applied Dataset-1 and Dataset-2, from Kaggle website respectively.

Abdulmunem, Abutiheen & Aleqabie (2021) presents a methodology to recognize COVID-19 cases using Resnet-50 with 5 and 10 folds cross validation and the resulting accuracy reached to 97.28%.

Ismael & Şengür (2021) proposes a pre-trained CNN model to extract and classify CXR images to detect COVID-19 cases further, they used Resnet-50 and SVM with linear kernel function and they obtain a satisfied accuracy reached to 94.7%. They used limited number of CXR images and therefore a suggestion of performing augmentation based on limited nimber of imbalanced data is required.

A new self-contained dataset for COVID-19 classification is presented by Misztal et al. (2020) by which they used number of CT and radiograph images from a diverse set of classes. Dense-Net is applied to CT with radiograph and the resulting accuracies are 87% and 92% for multiclass and data stock binary, respectively. They suggested to use 3D CT images with radiograph to analysis and learn other patients on clinical.

Santosh (2021), summarizes artificial intelligence for COVID-19 issues ranging from forecasting to decision making to support healthcare in human life. Moreover, in Joshi, Dey & Santosh (2020) presented intelligent systems and methods to combat Covid-19.

We could conclude from the literature as mentioned earlier, the following points

- The importance of the deep learning models and the pre-trained-models for the early diagnoses of COVID-19.
- Regards to the limited available resources and COVID-19 X-ray images, we have employed the role of the data augmentation methods effectively in generating a lot of required images.
- Different types of clinical data (balanced and imbalanced) with different features range and values should be used to test any proposed model related to COVID-19 to assure reliability and validity of the system.

## Materials and Methods

### Deep learning

Generally, deep neural network (DNN) inspired by the human brain consists of neurons, synapses, and much more, the formulation of DNN seems like hierarchical neural networks to improve the process of classifying supervised patterns (Hinton & Salakhutdinov, 2006; Ciregan, Meier & Schmidhuber, 2012). DL is a methodology of stacking multi-hidden layers that can significantly learn objects. DL can classify, extract the features, and make a decision ineffective and precise fashion after an efficient training process. The training process includes “fine-tuning” where DNN slightly adjusts the weights found in pre-training during backpropagation (Hinton et al., 2012; Yosinski et al., 2014). Hence, we need optimizers during parameters update and cost minimization. Mini-batch gradient decent (MBGD) and Adam optimizers are the most common optimizers to speed up the learning process and further enhance the value of the objective function. However, recently transfer learning plays a useful and powerful tool to enable the training of large-scale datasets without overfitting problem results from the target dataset that is much smaller than the raw dataset (Lu et al., 2015; Ahmed, Jones & Marks, 2015).

### MBGD and adam optimizers

The presence of redundancy of data is beneficial to use the MBGD as an optimizer algorithm. Since the learning rate changes from relatively large to the relatively small that is called schedule. Therefore, it is requiring to estimate the parameters to be convergence by futzing the parameters, ultimately (Le et al., 2011). The stochastic gradient descent (SGD) is used to minimize the objective function for extensive training sets. The traditional gradient descent becomes a costly procedure (Ruder, 2016). Assume that the hypothesis ${h_{\rm \theta}}\left( x \right)$ for *x* input features is given by Eq. (1) (Dean et al., 2012; Maas, Hannun & Ng, 2013).

(1) $${h_{\rm \theta}}\left( x \right) = \mathop \sum \limits_{j = 0}^{n} {{\rm \theta}_{j}}{x_{{j}}},$$

where $\rm \theta$ is the current initial case for the input *x* for the *n* training set, then the training set of hypothesis *h* called$\; {J_{\rm train}}\left({\rm \theta} \right)$ is given by half number of the *m* training examples multiplied by the average square error, as given in Eq. (2).

(2) $${J_{\rm train}}\left({\rm \theta} \right) = \displaystyle{1 \over {2m}}\mathop \sum \limits_{i = 1}^{m} {\left( {{h_{\rm \theta}}({x^{\left({i} \right)}}) - {y^{\left({i} \right)}}} \right)^{\rm 2}}$$

where the difference, ${\left( {{h_{\rm \theta}}({x^{\left({i} \right)}}) - {y^{\left({i} \right)}}} \right)^2}$, is the square error of the *i*th training instances such that the parameters of theta ${\rm \theta}$ in the inner loop of the gradient descent are updated repeatedly as in Eq. (3), and that is commonly called Batch Gradient Descent (BGD).

(3) $${{\rm \theta}_{j} = {{\rm \theta}_{j}} - \alpha \displaystyle{1 \over m}\mathop \sum \limits_{i = 1}^{m} {\left( {{h_{\rm \theta}}({x^{\left({i} \right)}}) - {y^{\left({i} \right)}}} \right)}\; {x_{j}}^{\left({i} \right)}}$$

where $\alpha \;$ is the learning rate for *j* = 0, 1, 2, …, *n*, the updated theta ${\rm \theta}$ is the difference between the old theta $\theta$ and the partial derivative ${\partial  \over {\partial \theta }}{J_{{\rm{train}}}}\left( \theta  \right)$. The parameters are firstly initialized, then different iteration of the batch gradient descent will likely result in local minimum through the data trajectory. For a large scale, data BGD accumulates the sums, and it will consume much time, and the system complexity will be very high. Therefore, both SGD and MBGD is more reliable than BGD in large scale optimization approaches, and with the systems requires different parameters. Moreover, there is no need, to sum up, all the training sets. Mini-batch Gradient Descent looks at the mini-batch instances. Also, SGD is more likely to escape from local optima than BGD, not guaranteed global optima. As the MBGD takes the batch reasonability straight-line trajectory to get the local minimum. The cost of SGD is given by Eq. (4) such that for random shuffle dataset the trained ${J_{\rm train}}\left({\rm \theta} \right)$ is the sum of the cost function to the number of the trained examples m, as shown in Eq. (5). Hence, the updated ${\rm \theta}$ does not depend on the accumulated summation, as shown in Eq. (6). During the learning process, it is preferred to use the largest number of iterations for optimizing the accuracy of the model. Starting from the first iteration may take the parameter in the direction and move the parameters in the direction of the local minima (Abadi et al., 2016; Lee et al., 2011). While BGD used all m examples in each iteration, and SGD used a single example in each iteration, the MBGD uses b examples in each iteration such that b is the mini-batch size as shown in Eq. (7) (Hinton, Srivastava & Swersky, 2012; Goyal et al., 2017; Jain et al., 2017). Moreover, Adam optimizer presented by Kingma & Ba (2014) depends on optimizing lower-order moments with a little memory requirement is applied in this work to boost the cost function as well as to get reliable results after MBGD. A mathematical proof of the convergence Adam optimizer is stated in Tran (2019).

(4) $${\rm cost}\left( {{\rm \theta} ,({x^{\left( {i} \right)}},{y^{\left({i} \right)}})} \right) = \displaystyle{1 \over 2}{\left( {{h_{\rm \theta}}({x^{\left({i} \right)}}) - {y^{\left({i} \right)}}} \right)^{\rm 2}}$$

(5) $${J_{\rm train}}\left({\rm \theta} \right) = \displaystyle{1 \over m}\mathop \sum \limits_{i = 1}^{m} {\rm cost}\left( {{\rm \theta} ,({x^{\left({i} \right)}},{y^{\left({i} \right)}})} \right)$$

(6) $${{\rm \theta}_{j}} = {{\rm \theta}_{j}} - \alpha \left( {\left( {{h_{\rm \theta}}({x^{\left({i} \right)}}) - {y^{\left({i} \right)}}} \right)\; {x_{j}}^{\left({i} \right)}} \right)$$

(7) $${{\rm \theta}_{j}} = {{\rm \theta} _{j}} - \alpha \displaystyle{1 \over b}\mathop \sum \limits_{i = 1}^{b} \left( {\left. {{h_{\rm \theta}}{{\left( x \right.}^{\left({i} \right)}}} \right) - {y^{\left({i} \right)}}} \right)\; {x_{j}}^{\left({i} \right)}$$

### Generative adversarial networks (GANs)

Generative Adversarial Networks (GANs) are recently used for the generation of images, video, and voice. GANs are algorithmic architectures that use two DNN architectures, to build new simulated instances of data that can be transferred to real data (Goodfellow et al., 2014). GAN is one of the most common architecture algorithms for image data augmentation by which the samples can be stacked with random cropping to further in-rate the data collection (Samangouei, Kabkab & Chellappa, 2018; Frid-Adar et al., 2018).

**Figure 1:**
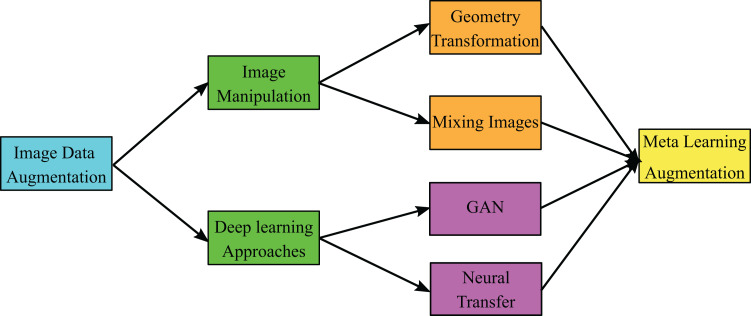
General taxonomy of image data augmentation.

In Waheed et al. (2020) the authors presented GAN architecture for a limited number of COVID-19 CXR dataset that consists of 192 images and their architecture achieved accuracies 85% and 95% before and after GAN augmentation, respectively. The taxonomy of image data augmentation that shows the different types recently used in image augmentation is shown in Fig. 1 (Shorten & Khoshgoftaar, 2019). In image data augmentation, there are two types; the first type is image manipulation, while the second is DL approaches. In image manipulation, there are two types in general, which are geometry transformation and mixing images. In contrast, the DL approaches are classified into GANs and neural transfer. In this work, we present GAN architecture, which belongs to DL approaches to produce meta-learning data augmentation of the enrolled CXR images.

### The proposed CXRVN architecture

**Figure 2:**
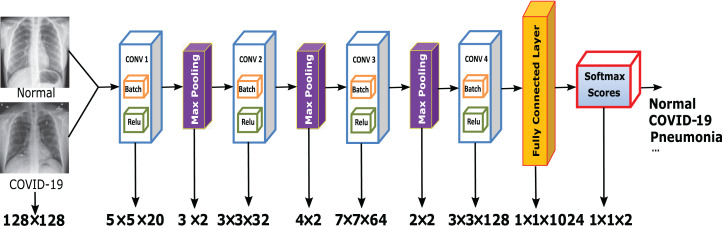
The proposed CXRVN architecture for COVID-19 classification using fully connected DCNN. The CXR images were downloaded from Kaggle under a CC0 1.0 license.

In this article, we build a novel architecture to classify the input COVID-19 CXR images into normal and abnormal categories. The proposed network is called Chest X-Ray COVID-19 Network (CXRVN) is considered as the first specialized deep neural network for analyzing chest X-ray images against the pandemic COVID-19. Our network architecture is summarized in Fig. 2. Generally, CXRVN consists of four convolution layers, three pooling layers, and one fully connected layer. Next, we describe the main features of our architecture and their importance for diagnosis COVID19 patients.

Since the saturating nonlinearities are much slower than the non-saturating nonlinearity during the training time with gradient descent, we use the rectified linear unit (ReLU) as the activation function. Besides, we concern about preventing overfitting when using ReLU, so the observed effect is different from the accelerated ability to fit the training set. Besides, using ReLU during the training procedure leads to relatively rapid learning of the network.

The ReLU is the activation function used in the hidden layer so that for the input convoluted feature x, the ReLU allows faster learning, which significantly affects the output of large models trained on large datasets, and does not require normalization of inputs to avoid saturation of the learning.

Although some training examples produce a positive input to a ReLU of a neuron, causing the learning process to happen in that neuron, we still find the importance of local normalization scheme in generalization aids. The batch normalization that determines the mean and variance for the input feature x is determined as in Calik et al. (2019) by which the mean of the expected value of x is determined. Moreover, the variance is the expected value the determined as the square of each enrolled features *x* subtracted from the mean of the whole features $\mu$. Then the normalized value of *x* is calculated by Eq. (8) as follows:

(8) $$\dot x = \; \displaystyle{{{x_{i}} - {\rm \mu}} \over {\sqrt {{\sigma ^2} -{\in}} }}$$

where $\in \;$ is a very small number which protects zero division for batch normalization via deep learning platform codes.

For the score vectors of an input COVID-19 X-Ray input images that denoted by ${S^{{\rm V_x}}}$, the probability of the scored values are given as in Eq. (9)

(9) $${P_i} = \displaystyle{{{e^{{S^{{V_x}}}}}} \over {\mathop \sum \nolimits_{i = 1}^n {e^{{S^{{V_i}}}}}}}$$

Typically, we find that models with concurrent pooling are much more challenging to overfit during the training models. Because of the pooling layers in our CNNs, the architecture summarizes the outputs in the same kernel map of neighboring groups of neurons. A pooling layer can, therefore, be viewed as a grid of pooling units spaced between pixels, each of which summarizes a neighborhood of size *z* × *z* centered at the pooling unit position. The use of overlapping pooling layers decreases error levels compared to the non-overlapping system, which generates equal dimensional outputs.

In the proposed architecture, we use four convolutional layers. Each layer contains a batch normalization and ReLU activation function. The batch normalization is applied for the mini-batch set of the learned parameters of the convoluted features so that it brings the mini-batch data to zero means and normalizes variance.

For the abnormal cases that required the percentage of the existence of the COVID-19 virus inside the image, the proposed system determines the abnormal probability infection using the SoftMax activation function. The algorithm steps of the proposed CXRVN is shown in Algorithm 1.

**Algorithm 1:** Build a deep learning model using CXRVN-proposed architecture.

Input $\leftarrow$ Image_COVID-19_Set imds
Output $\leftarrow$ CXRVN
Begin // Preprocessing COVID-19 X-Ray image(s) in imds For i=1: length(imds) img $\leftarrow$ read(imds,i) cxr $\leftarrow$ isXGray(img) img $\leftarrow$ resize(cxr,[128, 128]) save(imds,I,img) End for // Build CXRVN Structure NLayers $\leftarrow$ new Layers{} NLayers.append(new Input layer) NLayers.append(new Convolutional layer) NLayers.append(new Normalization layer) NLayers.append(new Relu layer) NLayers.append(new Pooling layer) NLayers.append(new Convolutional layer) NLayers.append(new Normalization layer) NLayers.append(new Relu layer) NLayers.append(new Pooling layer) NLayers.append(new Convolutional layer) NLayers.append(new Normalization layer) NLayers.append(new Relu layer) NLayers.append(new Pooling layer) NLayers.append(new FeatureConnected layer) NLayers.append(new Softmax layer) NLayers.append(new Classification layer) // Train CXRVN using options Options.set(SolverOptimizer $\leftarrow$ mini-batch gradient decent with momentum or Adam) Options.set(InitialLearnRate ←1e-3) Options.set(LearnRateSchedule ← Piecewise) Options.set(MiniBatchSize ←32) Options.set(LearnRateDropFactor ←0.2) Options.set(LearnRateDropPeriod ←5) Options.set(Shuffle←Every Epoch) Options.set(ValidationFrequency←2) Options.set(MaxEpochs ←20) CXRVN $\leftarrow$ trainNetwork(NLayers, imds, Options) End

We train our models using MBGD with a mini-batch size b=32 and a weight decay of 0.0003. This small amount of weight decay is essential for practical training as it is not only a regularization strategy but also it reduces the model’s training error. Furthermore, we apply Adam optimizer based on the same hyper-parameters to boost and ensure the system’s reliability in speed computation time and minimum memory.

On the other hand, we adopt GANs to construct artificial instances for further data augmentation. In Algorithm 2, we summarize the steps of the proposed trained GAN augmentation model.

**Algorithm 2:** Build a deep Learning model using CXRVN-proposed architecture.

Input $\leftarrow$ Image_COVID-19_Set imds, Generator Layers GeLayers, Discriminator Layers DiLayers
Output $\leftarrow$ Generator GEN, Discriminator DISC
Begin // Setting up Training options Options.set(ValidationFrequecy $\leftarrow$ 5) Options.set(InitialLearnRate ←1e-4) Options.set(LearnRateSchedule ← Piecewise) Options.set(MiniBatchSize ←16) Options.set(MaxEpochs ←50) GAN $\leftarrow$ LGraph2Net(GeLayers) DISC $\leftarrow$ LGraph2Net(DiLayers) // Training GEN and DISC using imds For i=1: MaxEpochs batchImgs $\leftarrow$ read(imds,BatchSize) Imgs $\leftarrow$ Shulfe(batchImgs) latentIN $\leftarrow \;$gen(Imgs,GEN) DPred $\leftarrow$ Forward(DISC, latentIN) GPred $\leftarrow$ Forward(GEN, latentIN) DProb $\leftarrow$ Sigmoid(DPred) GProb $\leftarrow$ Mean(DProb) Loss $\leftarrow$ CalcLoss(DProb, GProb) GAN $\leftarrow$ CalcGradients(GAN.Learnables,Loss) DISC $\leftarrow$ CalcGradients(DISC.Learnables,Loss) End for End

The excellent success of GANs has led to an increased focus on how they can be applied to the data increment mission. In this article, we take the real images in the dataset, and the discriminator compares it with the generated images represented by the applied noise that represents the difference of the enrolled real images to predict the labels. The generator is applied as input to a given vector of random values (latent inputs), and the network generates data with the same structure as the training data. Discriminator, given batches of data containing both the training data and the generated data from the generator, is trying to classify the observations as “real” or “generated.” Training to produce data that “fools” the discriminator. Train the discriminator to differentiate between real and produced data (Wang, Wang & Wang, 2018; Salehinejad et al., 2018). Due to the limited and variable number of the collected database from different sources, in this work, we collected a database and made GAN augmentation available on the Mendeley website (Shams et al., 2020a).

As we present a CXR COVID-19 Network CXRVN that is build from scratch to identify, recognize and classify chest X-ray images in a simple and very fast manner. Moreover, the proposed methodology used GAN network to augment the limited number of imabalanced dataset from different sources and handled it in an efficient and reliable manner. The comparison of the proposed method compared with the state of the art approaches stated the reliability of the proposed system in terms of accuracy and loss function before and after GAN augmentation.

### Evaluation of experimental results

This section is dedicated to exploring the effectiveness of the proposed approach. Due to the variability of the updated standard datasets versions COVID-19 X-ray images, two different experimental studies are carried out, discussed, and analyzed in detail.

All experiments were carried out using the MATLAB 2019b software package running on Microsoft machine with Core i7 processor, 16-RAM, and NVIDIA 4G- GT 740m GPU environment.

### Dataset characteristics

There are three datasets we are used in this article; the first one is called Dataset-1 from (Faizan, 2020) which contains 25 normal cases (negative cases) and 25 COVID-19 (positive cases). Smfai presents 50 images and he claims that COVID-19 cases reached to 100% recognized at a time and 80% for negative cases or normal cases. The second one named Dataset-2 from (Mooney, 2020; Bachir, 2020). It is noticed that Dataset-2 collected from two different independent sources, the first one Paul moony (Mooney, 2020) presented 5,863 chest x-ray images with two class labels pneumonia and normal cases. The collected chest-x-ray images also known as (posterior-anterior) were selected from retrospective cohorts of pediatric patients of one to five years old from Guangzhou Women and Children’s Medical Center, Guangzhou. All chest X-ray imaging was performed as part of patients’ routine clinical care. We used only 234 normal cases selected from 1,341 image with percentage (17.44%) and 148 pneumonia cases out of 3,875 trained cases. The second one is which has 221 COVID-19 cases selected from 314 images with percentage (70.38 %). The dataset is collected from 205 male and female patients with ages in between 120 and 88 years old.

The third dataset called Dataset-3 was uploaded in Menedely (Shams et al., 2020a). This collected data contains 603 chest-x-ray images with three class labels normal, COVID-19, and pneumonia cases which are 234, 221 and 148 respectively. We further augment the dataset using GANs, which produce 6,030 images; 2,340, 2,210, 1,480 for normal, COVID-19, and pneumonia images, respectively. The details of all datasets are summarized in Table 1. We used the dataset collected by Toğaçar, Ergen & Cömert (2020) for comparison study.

**Table 1:** The collected datasets of the normal, COVID-19, and pneumonia X-ray images before and after the augmentation process.

Dataset name	# of Instances	# of Classes	Labels	Balance
Dataset-1	50	2	Normal: 25	1.0
			COVID-19: 25	
Dataset-2	455	2	Normal: 234	0.94
			COVID-19: 221	
Dataset-3	603	3	Normal: 234	0.8
			COVID-19: 221	
			Pneumonia: 148	
Toğaçar, Ergen & Cömert (2020)	458	3	Normal: 295	0.7
			COVID-19: 65	
			Pneumonia: 98	

### Parameters optimization

In this part, we attempt to optimize the proposed CXRVN architecture using the Bayesian algorithm to minimize the scalar objective function. We need to update the Gaussian process model to find a new point that is required for maximizing the acquisition function by sampling thousands of pints with the variable bounds. Therefore, in this work, we propose to use MBGD to find the local minima that satisfy constraints. Moreover, the proposed CXRVN architecture is fitted to overcome the additive noise with minimum loss after GAN augmentation using both MBGD and Adam optimizers.

All experiments were done based on the following parameters. The hyper-parameter values of the proposed DCNN architecture, as shown in Table 2.

**Table 2:** Hyper-parameter values of the proposed CXRVN architecture.

Parameter	Value
Learning Rate	0.01
Batch Size	32
Momentum	0.8
Weight Decay	0.0003
Max no. of iterations	600

### Performance measures

To evaluate the performance of the proposed DCNN architecture, the well-known performance measures for the evaluation are used, in terms of, the sensitivity, specificity, precision, accuracy and F1score from the confusion matrix based on the following Equations:

(10) $$\rm Sensitivity = TP/ (TP+FN)$$

(11) $$\rm Specificity = TN/ (TN+FP)$$

(12) $$\rm Precision = TP/ (TP+FP)$$

(13) $$\rm Accuracy = (TP+TN)/ (TP+TN+FP+FN)$$

(14) $$\rm F1-score= 2TP/ (2TP+FP+FN)$$

where TP, TN, FP, and FN are true positive, true negative, false positive, and false negative, respectively.

### Evaluation of the proposed CXRVN

The evaluation of the proposed CXRVN was performed to get the final decision of the trained model. Initially, the datasets, which contain the normal, COVID-19, and pneumonia cases, are collected and enrolled. Afterward, the datasets are splitted into training and testing sets. The trained images are then applied to the GAN augmentation process, which takes the trained sets of the enrolled image and generates the synthesized image datasets to produce an augmented COVID-19 dataset.

**Figure 3:**
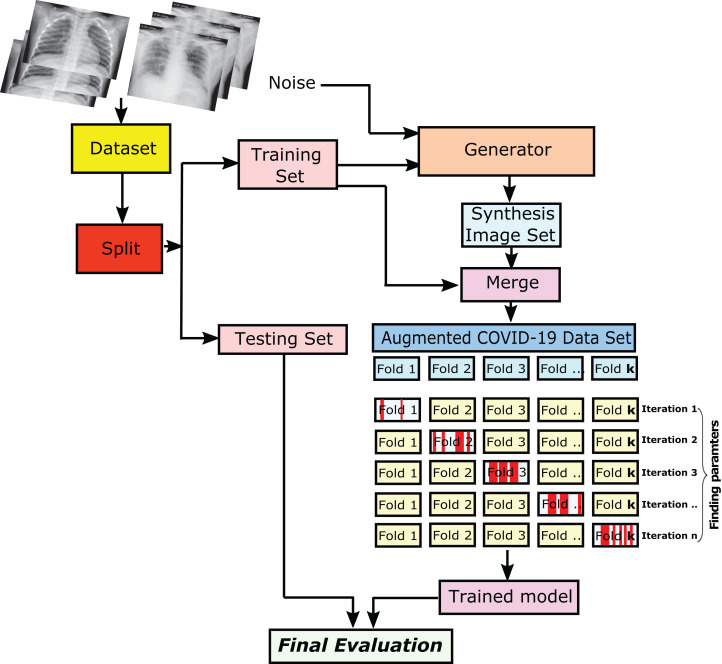
The steps of getting the final evaluation results of the trained/tested augmented COVID-19 chest X-ray images. The CXR images were downloaded from Kaggle under a CC0 1.0 license.

We used the *k*-fold cross-validation strategy with *k* = 10 cross validation. Specifically, we train on *k−*1 folds and validate on the remaining 1-fold. Then we calculate the average from *n* iterations as shown in detail in Fig. 3. We able to control the number of iteration of the trained COVID-19 chest X-ray images to be compared with tested images to obtain the final evaluation.

The collected datasets consist of normal cases and COVID-19 ones. These datasets are splitted into two sets, and they are the training and testing sets. To overcome the overfitting problem, We split the datasets into 80% for the trained images, and the remaining 20% is for the testing ones. Subsequently, the training sets are augmented via the use of GAN. Therefore, the hyper-parameters values of the training sets have learned and proceeded with the evaluation to produce the validation set. Every iteration of the shuffled fold is split by generating an independent number of the trained/tested image.

### Experiment (I): Dataset-1

**Figure 4:**
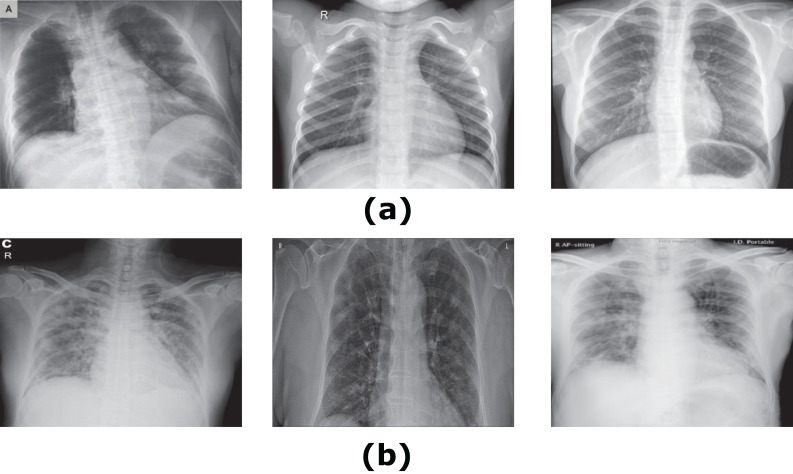
Samples of Dataset-1 (Faizan, 2020). (A) Normal X-Ray images, (B) infected COVID-19 X-Ray images. The CXR images were downloaded from GitHub under a CC0 1.0 license.

**Figure 5:**
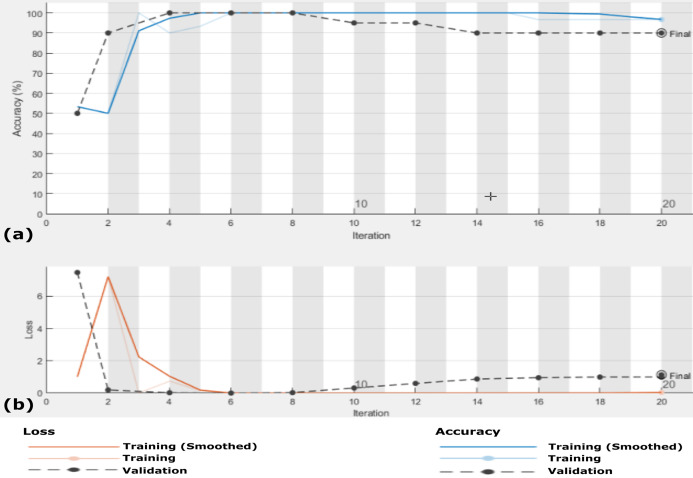
Accuracy and loss of the proposed CXRVN architecture for the testing X-ray images in Dataset-1 (A) Accuracy reaches 88% of the testing images, (B) loss enhancement after 20 iteration.

The first experiment was conducted using dataser-1. This experiment is performed using the same parameters mentioned in Table 2. Subsequently, we used the proposed architecture shown in Fig. 2 by enrolling all 50 grayscale images to the system. The enrolled images are with size 128 × 128 × 1. Furthermore, we used the mini-batch gradient descent optimizer for the trained convoluted input features. Afterward, the maximum pooling of the convoluted images to produce the fully connected layer that contains 1 × 1 × 1,024. Finally, the CXRVN classify the results either to normal or COVID-19 case. A sample of the dataset is shown in Fig. 4. This experiment is considered as an initial experiment to prove the ability of the proposed architecture to handle and solve the classification problem based on the small collected dataset. The accuracy and loss function of the proposed CXRVN architecture for a given hyper-parameter value is shown in Fig. 5.

**Figure 6:**
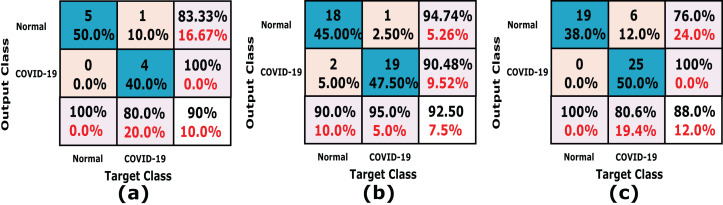
A detailed confusion matrix results of the proposed CXRVN system. (A) Testing (20%). (B) training (80%) (C) 10-fold Cross Validation.

In this experiment we used *k*-fold cross-validation (*k* = 10) for all stacked 50 X-ray images. The confusion matrices of the achieved results are shown in Fig. 6, which show that the proposed system accuracy achieved for testing, training, and cross-validation are 90.0%, 92.5% and 88%, respectively. The confusion matrix measurements, including sensitivity, specificity, accuracy, precision and *F*1 score in both cross validation, training, and testing phases are summarized in Table 3. We notice that, the average results of the proposed CXRVN architecture is 92.85% in the testing phase.

**Table 3:** Performance measures of the proposed model on Dataset-1.

Dataset-1			
CXRVN-architecture	Validation Methodology		
	Cross Validation	Holdout	
	10-fold cross validation (%)	Training (80%)	Testing (20%)
Sensitivity	76.00	94.74	83.33
Specificity	100.00	90.48	100.00
Precision	100.00	90.00	100.00
Accuracy	88.00	92.50	90.00
F1score	86.36	92.31	90.91
Average	90.07	92.01	92.85

Intuitively, only 50 X-Ray images are not sufficient to prove the reliability and robustness of the proposed system. Therefore, we have to expand the data of the X-ray COVID-19 images. Furthermore, enhancement of the augmentation process by applying GAN augmentation for the input images is urgently required.

### Experiment (II): Dataset-2

**Figure 7:**
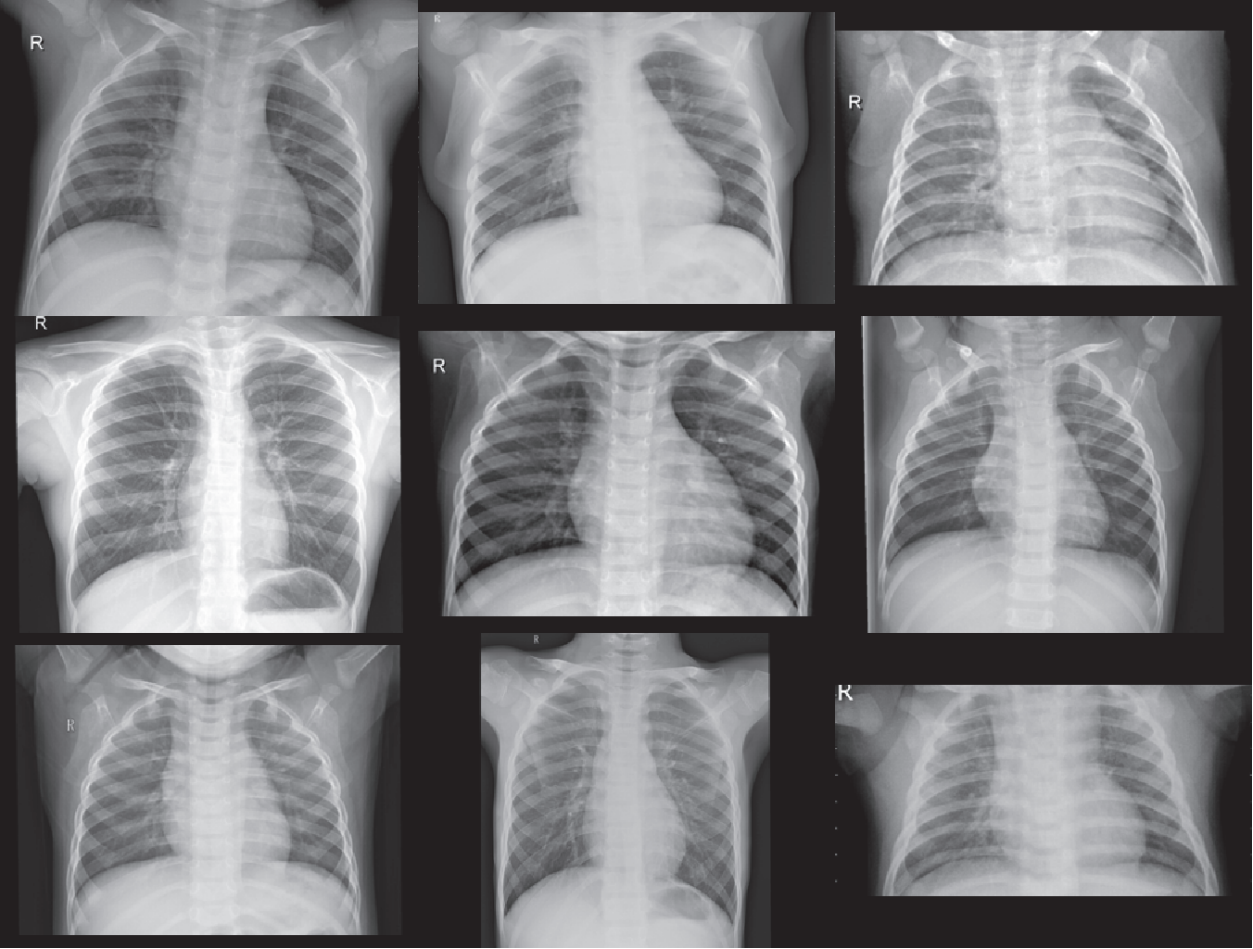
Normal Cases out of Dataset-2 from (Mooney, 2020). The CXR images were downloaded from Kaggle under a CC0 1.0 license.

**Figure 8:**
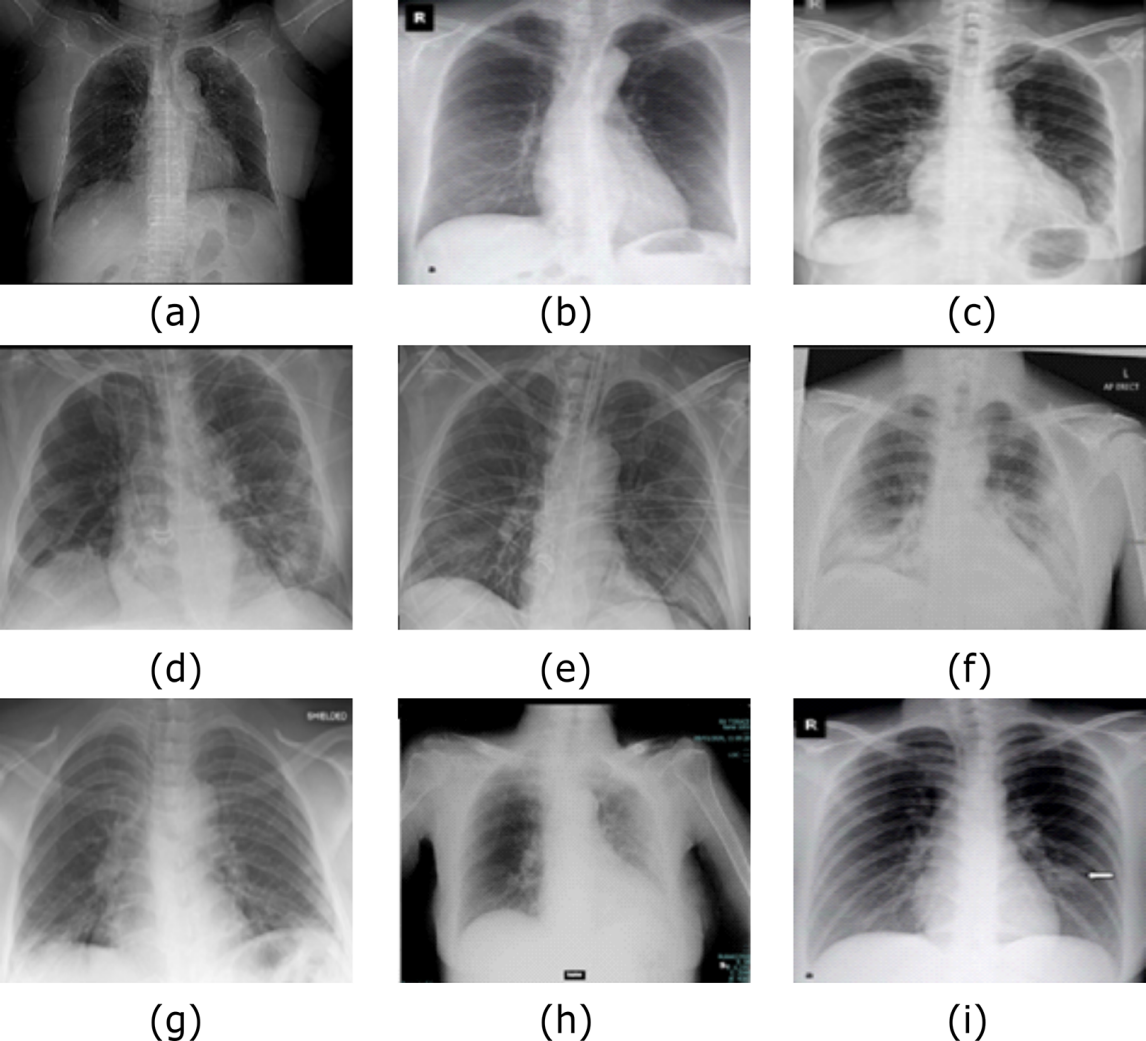
Samples of COVID-19 Cases (A–I) out of Dataset-2 from (Bachir (2020). The CXR images were downloaded from Kaggle under a CC0 1.0 license.

In this experiment, we utilize Dataset-2 based on the same hyper-parameter values mentioned in Table 2 for normal and COVID-19 X-ray images. Furthermore, for image data augmentation, GAN is used in the preprocessing stage to ensure the reliability of the proposed system and to enhance the results in a large-scale standard dataset with the same hyper-parameter values. The validation is processed every two iterations; also, every ten cycles, the data is shuffled (10-fold cross validation). In this experiment, afterward, the data shuffled and using MBGD, the regulator rate is 0.0001 given that the number of the trained images is 364, and the number of validated images 91, and we used the evaluation results based on 80% training and the remaining 20% for testing. Figures 7 and 8 show the samples of the dataset used (Mooney, 2020, at https://www.kaggle.com) and (Bachir, 2020, at https://www.kaggle.com) respectively.

**Figure 9:**
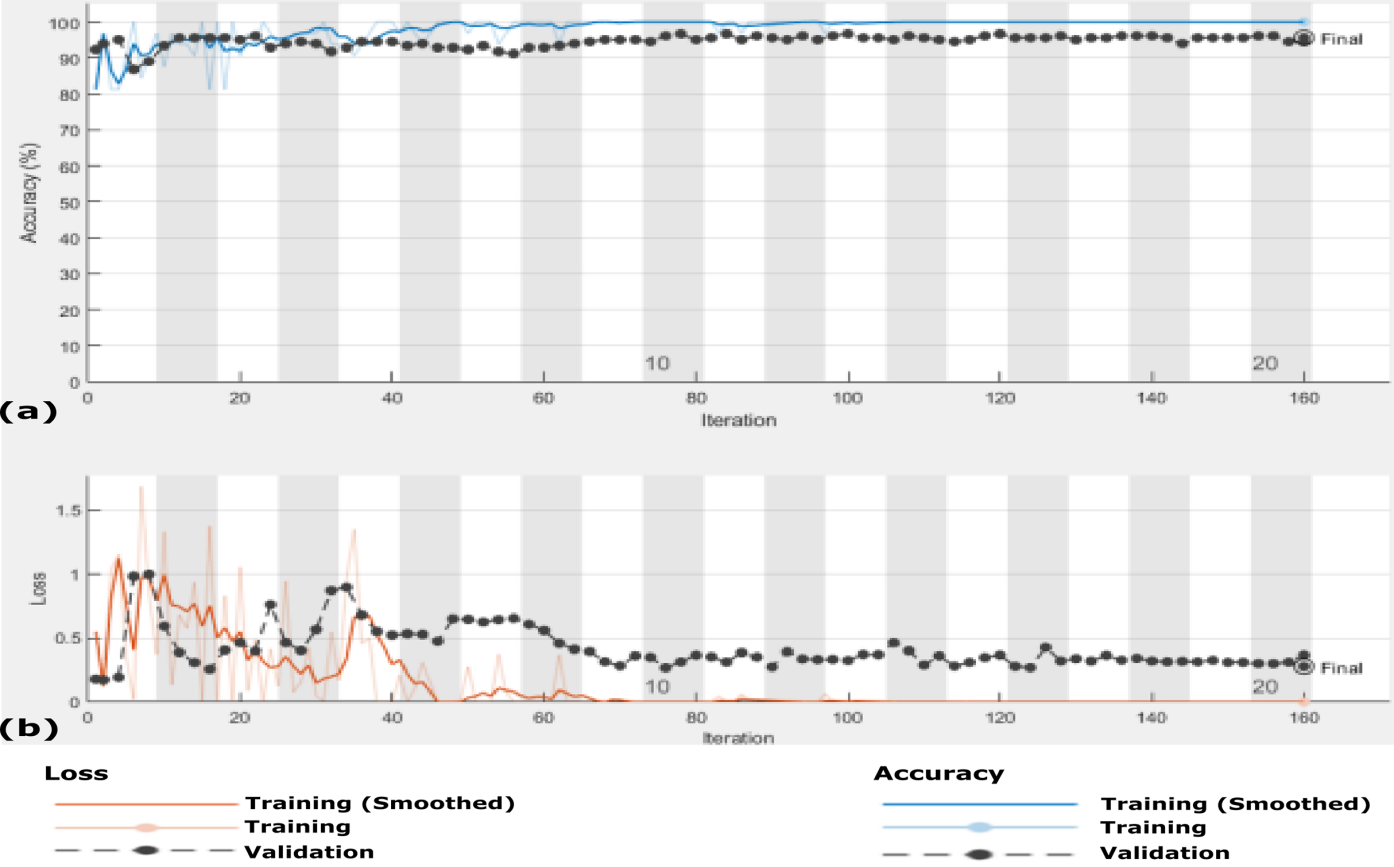
The accuracy and loss of the proposed CXRVN architecture for scenario 1 in Dataset-2 without GAN augmentation in testing. (A) The accuracy (96.70%) and (B) loss after 160 iteration.

We perform this experiment in two scenarios. The first scenario is using Dataset-2 without augmentation, that is, using only 455 images. The second scenario is after GAN augmentation, which generates 4,550 images based on the hyper-parameter values listed in Table 2. The results of the two scenarios are summarized in Table 4, which prove the system reliability. The CXRVN-architecture has critical advantages, including the reliability and stability of the running process. During changing the datasets, the CXRVN shows the solidity of architecture against a source of dataset and size. The accuracy and loss function of the proposed system architecture for phase 1 without data image augmentation is shown in Fig. 9. In this experiment the final result that represents the accuracy of the tested 91-X-ray images is 96.70% after 160 iteration. It is very clear that, the loss function is slightly high after 20–40 iteration and it becomes more stable with minimum value after 160 iteration. In this experiment we used MBGD optimizer before augmentation with *b* = 32. Therefore, we need to enhance the loss and accuracy at the same time. To boost the results obtained and produce enhancement accuracy with a minimum loss, Adam optimizer with MBGD is applied in the X-ray image augmentation based on GAN architecture.

**Table 4:** The collected datasets of the normal, COVID-19 and pneumonia X-ray images before and after the augmentation process.

Dataset-2		
CXRVN-architecture	Scenario 1 without augmentation (%)	Scenario 2 augmentation using GAN (%)
Sensitivity	97.83	98.91
Specificity	95.56	96.24
Precision	95.74	96.38
Accuracy	96.70	97.58
F1score	96.77	97.63
Average	96.52	97.35

#### Scenario 2

**Figure 10:**
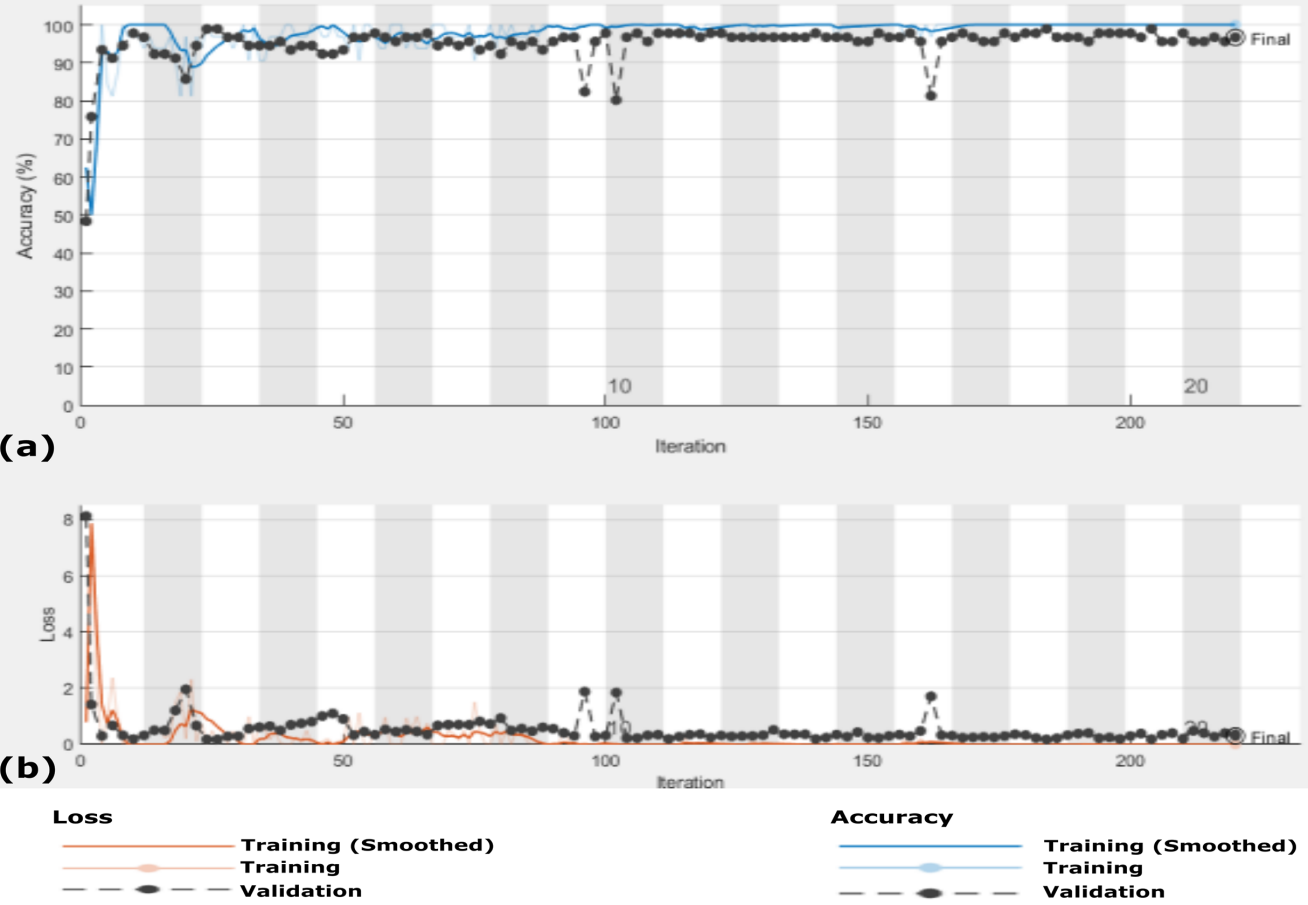
The accuracy and loss of the proposed CXRVN architecture for phase 2 After GAN augmentation in testing phase. (A) The accuracy reached to 97.35% and (B) the loss after 220 iterations.

In this scenario, we use the same 455 X-ray images that is, Dataset-2 and the data are enlarged 10 times to generate 4,550 X-ray images using GAN augmentation architecture. In this experiment, same hyper-parameter values in Table 2 are used but with MBGD instead of Adam optimizer, and the maximum number of iteration was 220. Figure 10 shows the accuracy and loss function of the proposed CXRVN architecture on the 4,550 X-ray images after GAN augmentation using Adam optimizer.

**Figure 11:**
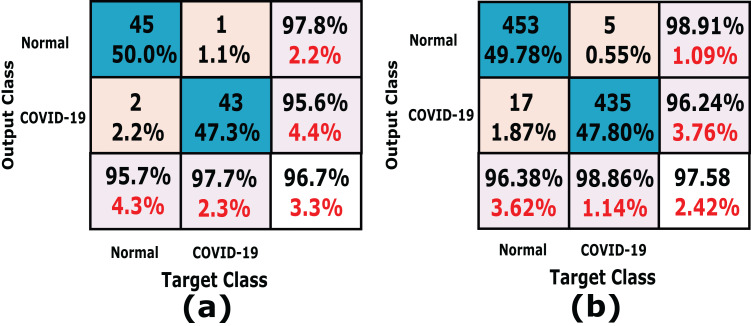
A detailed confusion matrix results of the proposed CXRVN architecture for experiment 2 applied on Dataset-2. (A) A total of 91 tested X-ray out of 455 images before augmentation for two classes normal, and COVID-19, respectively. (B) A total of 910 tested X-ray out of 4,550 images after GAN augmentation for two classes normal, and COVID-19, respectively.

It is noteworthy that, high accuracy of 97.58% is obtained after GAN augmentation with a minimum and stable loss after 220 iterations. Further, we prove the ability of the proposed CXRVN in the presence of big datasets of normal and COVID-19 X-Ray images. The confusion matrices of the two scenarios with and without data augmentation are shown in Fig. 11 for 91 and 910 tested X-ray images out of 455 and 4,550, respectively.

### Experiment (III): Dataset-3

**Figure 12:**
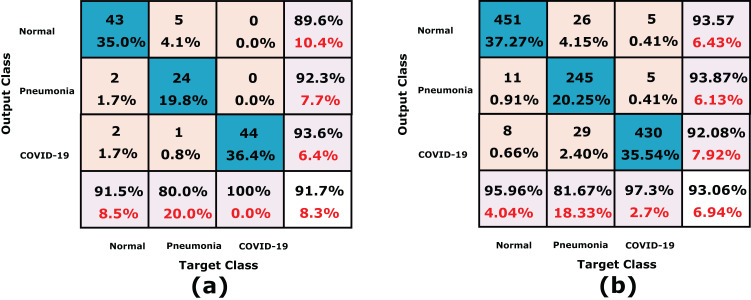
A detailed confusion matrices of the proposed CXRVN architecture for experiment 3 applied in Dataset-3. (A) A total of 91 tested X-ray out of 455 images before augmentation for two classes normal, and COVID-19, respectively. (B) A total of 910 tested X-ray out of 4550 images after GAN augmentation for two classes normal, and COVID-19, respectively.

In this experiment, the proposed CXRVN is evaluated based on three class labels as mentioned in details in Table 5. The data are collected from Dataset-2 except we modify 148 pneumonia datasets. The source of our collect dataset is uploaded on Mendeley website (Shams et al., 2020a). In this experiment the number of trained datasets are 482 and the remaining 121 are used for testing. The augmentation based on the proposed GAN architecture is performed to produce 6,030 augmented images 4,820 (80%) for training and 1210 (20%) for testing. The confusion matrixes of the three class labels are shown in Fig. 12A that shows the accuracy of our model which reaches 91.7% before augmentation.

**Table 5:** The collected dataset class distribution for experiment III on Dataset-3.

No	Class label	Raw dataset		Augmented GAN dataset	
		Train	Test	Train	Test
1	COVID-19	177	44	1770	440
2	Normal	187	47	1870	470
3	Pneumonia	118	30	1180	300
Total		482	121	4820	1210
		603		6030	

**Figure 13:**
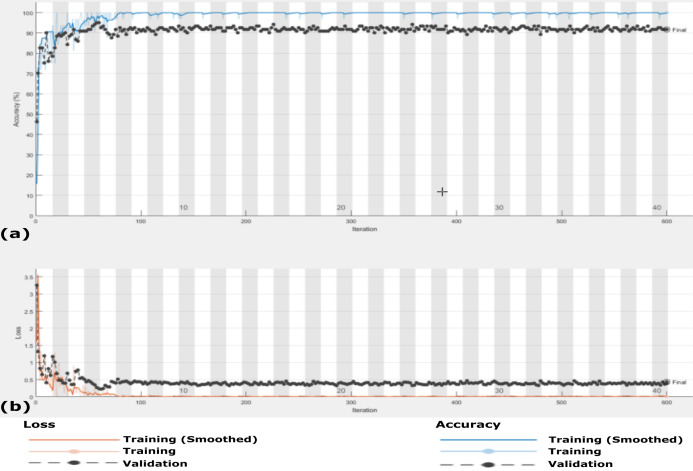
The accuracy and loss of the proposed CXRVN architecture for the three class label normal, pneumonia, and COVID-19. (A) The accuracy reached to 93.07% and (B) loss after 600 iterations and GAN augmentation.

On the other hand, the accuracy of the proposed CXRVN based GAN augmentation achieved improved accuracy of 93.06% on the tested 1210 augmented X-ray images for three classes as shown in Fig. 12B. It is obvious that, there is slight decrease in accuracy of the three class labels compared with the two classes. This is because the presence of three classes that collected from different sources that is, imbalance dataset. On the contrary, there is an improvement of the loss function as shown in Fig. 13. To improve the loss and classification accuracy we utilize GAN architecture for augmentation. Figure 12 shows the evaluation results related to experiment (III) of the three classes; COVID-19, normal, and pneumonia. It can be noticed that, a great enhancement of results after GAN augmentation is achieved by using Adam optimizer based on MBGD.

The accuracy and loss of the proposed CXRVN architecture based on GAN X-ray image augmentation are shown in Fig. 13. We notice that, the final result is 93.06% after 600 iteration based on Adam optimizer with mini-batch size 20. Moreover, enhancement and stability of the loss function during the experiment reach 600 iterations. We used the same parameter values listed in Table 2. Table 6 summarizes the recall, precision and accuracy of the proposed CXRVN architecture before and after GAN augmentation.

**Table 6:** Recall, precision, and accuracy of the two phases in experiment 3 (Dataset-3).

No	Class label	Before augmentation			After GAN augmentation		
		Recall	Precision	Accuracy	Recall	Precision	Accuracy
1	COVID-19	100	100.00	91.7	100	100	93.07
2	Normal	89.58	91.49		93.57	95.96	
3	Pneumonia	100	96.00		98.,00	89.42	

### Comparative analysis

To compare the proposed CXRVN architecture with the recent approaches, we need a normalized standard dataset. Moreover, that is not possible because of the variability and updated standard datasets for COVID-19 X-Ray images. Therefore, the proposed approach is firstly compared with the state-of-the-art deep learning models. Namely, the GoogLeNet, VGG-16, Resnet-18 and AlexNet. Although, these models are basically proposed for computer vision tasks, we made some modifications to be adaptive with the enrolled classes as a transfer learning models. The accuracy of the proposed method against these models on Datasets 2 and 3 are given in Table 7. This comparison is performed based on the same hyper-parameter values listed in Table 2 for databset-2. Moreover, we also perform a comparison on Dataset-3 that consists of three classes which are normal, COVID-19, and pneumonia. The comparison is performed based on the same hyper-parameter values in Table 2 except we used 40 epochs and the maximum number of iteration was 600. Table 7 summarizes the comparison evaluation in the testing phase for Dataset-3 based on three classes.

**Table 7:** Accuracy comparisons of proposed CXRVN architecture against Google net, VGG-16, Resnet-18, and Alex net on Dataset-2 and Dataset-3.

Method name	Class label	Google net	VGG-16	Res net-18	Alex net	Proposed CXRVN
Dataset-2 2-Classes	Normal COVID-19	92.20	90.75	93.20	91.10	97.85
Dataset-3 3-Classes	Normal COVID-19 pneumonia	91.01	89.35	91.65	92.21	93.06

**Figure 14:**
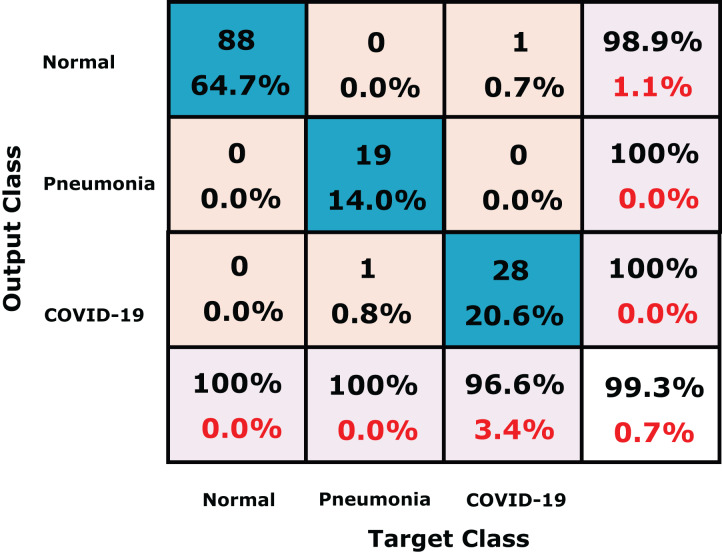
The confusion matrix of the dataset (Toğaçar, Ergen & Cömert, 2020) using CXRVN architecture.

We further compare our method the dataset collected by Toğaçar, Ergen & Cömert (2020) which consists of 458 chest X-ray images for three class labels; normal (65), COVID-19 (295) and pneumonia (98) shown in Table 8. For fair comparison with (Toğaçar, Ergen & Cömert, 2020), we used 70% and 30% for training and testing, respectively, as reported by the authors. The confusion matrix of our proposed CXRVN on (Toğaçar, Ergen & Cömert, 2020) dataset is shown in Fig. 14. Table 9 investigate the detailed comparison between (Toğaçar, Ergen & Cömert, 2020) and our CXRVN architecture.

**Table 8:** The collected dataset by Toğaçar, Ergen & Cömert (2020) class distribution.

No	Class label	Raw dataset	
		Train	Test
1	COVID-19	207	88
2	Normal	46	19
3	Pneumonia	69	29
Total		322	136
		458	

**Table 9:** The comparison between (Toğaçar, Ergen & Cömert, 2020) and our proposed CXRVN architecture.

Class label	Methodology	Accuracy (%)
COVID-19 Normal Pneumonia	SqueezeNet [18]	97.81
	MobileNetV2 [18]	98.54
	Proposed CXRVN	99.30

**Figure 15:**
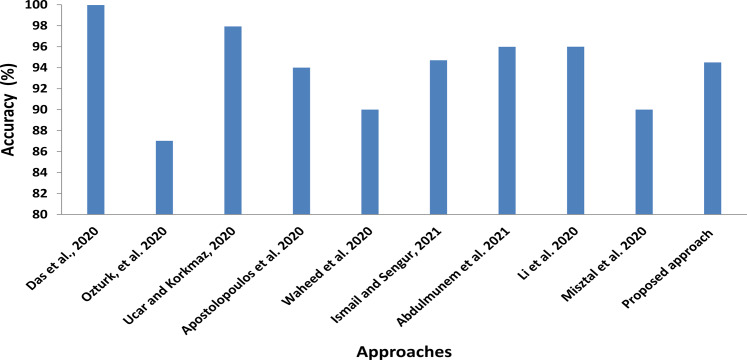
Statistical analysis of the average accuracy of the proposed approaches compared to the state-of-the-art methods.

In addition to comparisons with shared computer vision deep models, we also roughly compare our proposed CXRVN architecture with the state-of-the-art methods of COVID-19 detection and classification, as illustrated in Fig. 15 and Table 10. In this comparison, we show the class label, modality used, that is, X-ray and CT, number of cases, and the methodology used. Further, in Table 11 the running time for each scenario and dataset used are determined.

**Table 10:** Comparison study of the proposed CXRVN model and the state-of-the-art methodologies.

Author	Class label	Modality type of images	Number of cases	Methodology	Accuracy(%)
Das, Santosh & Pal (2020)	COVID-19 Pneumonia TB (China) TB (USA)	Chest X-Ray	162 4280 342 58	Truncated Inception Network	99.96
Ozturk et al. (2020)	COVID-19 Pneumonia No-finding	Chest X-Ray	125 COVID-19 500 Pneumonia 500 No-finding	DarkCovidNet	87.02
Ucar & Korkmaz (2020)	COVID-19 Pneumonia Normal	Chest X-Ray	76 COVID-19 4290 Pneumonia 1583 Normal	deep Bayes-SqueezeNet	97.93
Apostolopoulos, Aznaouridis & Tzani (2020)	COVID-19 Pneumonia Normal	Chest X-Ray	224 COVID-19 714 Pneumonia 504 Normal	MobileNet VGG-19	94.72 93.84
Waheed et al. (2020)	COVID-19 Normal	Chest X-Ray	72 COVID-19 120Normal	Before GAN After GAN	85.00 95.00
Ismael & Şengür (2021)	COVID-19 Normal	Chest X-Ray	95 End-to-end Training	Resnet-50	94.7
Abdulmunem, Abutiheen & Aleqabie (2021)	COVID-19 Normal	Chest X-Ray	25 COVID-19 25 Normal	Resnet-50	95.99
Li et al. (2020)	COVID-19 CAP Non- Pneumonia	Chest CT	1296 COVID-19 1735 Pneumonia 1325 Normal	Detection neural network (COVNet)	96.00 95.00 98.00
Misztal et al. (2020)	CT and Radiograph	Chest CT	Self-contained Dataset 6000	Dense net for data stock Dense net for multiclass	92.00 87.00
Perumal, Narayanan & Rajasekar (2020)	Bacterial Pneumonia Viral pneumonia Normal	Chest X-Ray, and CT	Bacterial Pneumonia 2,538 Viral pneumonia 1,345 Normal 1,349	Haralick+ VGG16, Resnet50 and Inception V3	93.8 89.2 82.4
Sahlol et al. (2020)	COVID-19 Normal	Chest X-Ray	Datasets 1 and 2 Kaggle.com	FO-MPA+ CNN	98.70 98.20 99.60 99.00
Proposed architecture	COVID-19 Normal	Chest X-Ray	Dataset-1 25 COVID-19 25 Normal	CXRVN	92.85
			Dataset-2 221 COVID-19 234 Normal	CXRVN	96.70
			Dataset-2- GAN augmentation 2210 COVID-19 2340 Normal	CXRVN	97.58
	COVID-19 Pneumonia Normal Chest X-Ray	Chest X-Ray	Dataset-3 221 COVID-19 234 Normal 148 Pneumonia	CXRVN	91.70
			Dataset-3- GAN augmentation 2210 COVID-19 2340 Normal 1480 Pneumonia	CXRVN	93.07

**Table 11:** The running time for each dataset and scenario of the proposed CXRVN model.

Classes	Modality	Dataset and scenario	Resulting accuracy	Running time
2 COVID-19 Normal	Chest X-Ray	Dataset-1 25 COVID-19 25 Normal	92.85	2 min 35 s
		Dataset-2 221 COVID-19 234 Normal	96.70	20 min and 50 s
		Dataset-2- GAN augmentation 2210 COVID-19 2340 Normal	97.58	45 min and 30 s
3 COVID-19 Pneumonia Normal	Chest X-Ray	Dataset-3 221 COVID-19 234 Normal 148 Pneumonia	91.07	80 min and 47 s
		Dataset-3- GAN augmentation 2210 COVID-19 2340 Normal 1480 Pneumonia	93.06	135 min and 58 s

## Discussion

The proposed approach is evaluated based two types of datasets, the first is Dataset-1 which is a balanced dataset, while the second is Dataset-2 which is an imbalanced one. For Dataset-1, we used only 50 cases; 50% normal and 50% are COVID-19 infected cases X-ray images. The results indicated that the accuracy of the proposed CXRVN is 92.85%. Furthermore, due to the variability and updated version of COVID-19 datasets, we collect a large-scale dataset from two sources for both normal and COVID-19. The GANs are used for image data augmentation to enlarge the collected datasets, and the experiment is performed in two phases.

Furthermore, two scenarios are presented, in the first one, which is done without augmentation, the accuracy reached to 96.70%, while the second one is based on the GANs augmentation, the accuracy reached to 97.58%. We not only used two class labels to validate the proposed method, but also we used three classes, including normal, COVID-19, and pneumonia. In experiment three applied in the collected Dataset-3 (Shams et al., 2020a), the accuracies are 91.07, and 93.06 before and after GAN augmentation, respectively. The comparisons between the proposed CXRVN and the most recent deep learning models are performed on our collected dataset, and the results indicate the superiority of our architecture. Moreover, we compare our method on the dataset collected by Toğaçar, Ergen & Cömert (2020) with the same parameter settings, and the results attain better performance, especially after GAN augmentation. Finally, rough comparisons between CXRVN and the state-of-the-art deep learning methods are performed and investigated in Table 10.

In Fig. 15 the statistical average values of the accuracy of the compared approaches with the proposed CXRVN average value. We clearly found that the average accuracy of the proposed approaches is not the top value that is because we used different imbalanced dataset than others. Moreover, we augmented the CXR images using GAN which increase the stability and accuracy of the proposed system (Shams et al., 2020b).

The technical motivation of the proposed CXRVN architecture can be summarized as follows:

- CXRVN classification architecture using the presence of chest X-ray images available allows diagnosis patients.
- The elapsed time to diagnosis the patients are decreased, and it will take a few seconds to classify the patient’s cases.
- The adaptability of the proposed CXRVN architecture in classification multi-class not only binary classes.
- In the proposed work, the model capable of handling large scale datasets by using GAN for augmentation.
- Mini-batch gradient descent and Adam optimizers are applied for GAN optimization.
- The available balanced and imbalanced of chest X-ray images are used as the input datasets.

We present a novel architecture that is trained from scratch with some modifications and improvements as follows:

- The network architecture is adaptive in its procedure parameter for the enrolled X-ray chest images.
- The architecture uses two optimizers for the augmented images, which are MBGD and Adam.
- The architecture can classify noisy X-ray images and produce promising layers as five convoluted layers with batch, and RelU activation functions are applied.
- The architecture deals with balanced, imbalanced, and augmented GAN datasets.
- The architecture can classify three classes normal, COVID, and pneumonia.

From the results mentioned above, it could be concluded the following points;

- Deep learning plays an essential role in detecting COVID-19 cases, smoothly.
- The role of GANs to produce different numbers of images helped to improve the overall accuracy of the proposed approach.
- The proposed approach would be used as transfer learning.

### Threads and limitations

Although the proposed method achieved superior performance to the state-of-the-art methods, it still has some limitations. The first one is that radiologists tested the accuracy of the proposed approach for clinical usage. The second limitation is the limited COVID-19 dataset, which is considered one of the most critical issues for training deep models. Using a big X-ray dataset for the training phase can potentially improve the performance of the proposed method. More extracted features of the X-ray images are required in order to test the X-ray images for more details that may be helpful for the updated cases in COVID-19 patients. Since CT scanners are not always available, usually have a high cost, and come after long acquisition time, X-ray remains the standard imaging modality for chest, particularly in isolated areas and developing countries.

### Future directions and Open challenge

There are different challenges related to the medical sector. They could be summarized in the following points. During the COVID-19 pandemic. Lots of challenges are faced

#### Dataset

Medical datasets are very limited, which is more difficult for any researcher to reach to these data. In the face of the epidemic Covid-19, the lack of images of the chest of various kinds. Researchers can use methods to enlarge the number of images, which contributes to a fair test of the different methods presented by researchers and research centers

#### Software

The diagnosis chest scan suffers the lack of ready programs for detecting the injury cases. Deep learning will play and still play an essential and vital role in contributing to the diagnosis of COVID-19 and others.

## Conclusions

Machine learning techniques, especially classification and regression, are considered as one of the essential tools to fight the spread COVID-19. In this article, a DCNN architecture to classify the input X-Ray COVID-19 images called CXRVN is proposed. The architecture can handle the extracted feature from each convoluted layer, and the results indicate the robustness and superiority of the proposed system compared with the state-of-the-art methods. We performed many different experiments based on availability and the applied dataset. The first experiment used a balanced dataset of 50 X-ray images for two classes (Dataset-1), normal and COVID-19, and the accuracy was 92.85% in the testing phase, while the second experiment was performed using an imbalanced dataset (Dataset-2) that consists of 455 X-ray images for two classes, and the accuracy was 96.70%. In the third experiment, we used 603 X-ray images for three class labels; COVID-19, normal, and pneumonia (Dataset-3), and the accuracy reached 91.70% in the testing phase. To prove the ability of the proposed CXRVN architecture on a large scale, we present image data augmentation based on GANs that leads to a significant enhancement of the proposed architecture. The evaluation results based on sensitivity, precision, recall, accuracy, and F1 score demonstrated that, after GAN augmentation, the accuracy reached 96.7% in experiment 2 (Dataset-2) for two classes and 93.07% in experiment-3 (Dataset-3) for three classes. Comparisons were performed to prove the robustness and reliability of the proposed architecture against the contemporary architectures. For future direction, we plan to use CT-images and study different updated cases of the COVID-19 X-Ray image. Furthermore, for the promising obtained results, the proposed architecture can be utilized in other medical images classification and diagnosis issues.

## Supplemental Information

10.7717/peerj-cs.358/supp-1 Supplemental Information 1 Code.

## Additional Information and Declarations

### Data Availability

The following information was supplied regarding data availability: Data is available at GitHub: https://github.com/smfai200/Detecting-COVID-19-in-X-ray-images/tree/master/dataset?fbclid=IwAR3UzNPQI9yfyETfbFVgCQHKWa5mvew5mJQxNhJk6tTYmLZsDzJZaDQ3bQ0. https://github.com/ieee8023/covid-chestxray-dataset/blob/master/README.md?fbclid=IwAR1AwKzp4dIY2WW_n1AQqTV0H9CDJ4GJQq6idffPmGBaFaKR6SWdwUBp78M. COVID-19 X-ray images are available at Kaggle: https://www.kaggle.com/bachrr/covid-chest-xray?fbclid=IwAR0KBoFoXsrv6LShModEDI1mcmVN0OEoLAg_7KKzcnHxiTH5huMBQApUMbc. Chest X-Ray (Pneumonia) images are also available at Kaggle: https://www.kaggle.com/paultimothymooney/chest-xray-pneumonia?fbclid=IwAR25LVOs7Wiu64v6bh_-SUZTEZKq1O3dM7xoZw5V1mqAk_Hx6gXjQfUv_GQ.

## References

[ref-1] Abadi M, Agarwal A, Barham P, Brevdo E, Chen Z, Citro C, Corrado GS, Davis A, Dean J, Devin M, Ghemawat S, Goodfellow I, Harp A, Irving G, Isard M, Jia Y, Jozefowicz R, Kaiser L, Kudlur M, Levenberg J, Mane D, Monga R, Moore S, Murray D, Olah C, Schuster M, Shlens J, Steiner B, Sutskever I, Talwar K, Tucker P, Vanhoucke V, Vasudevan V, Viegas F, Vinyals O, Warden P, Wattenberg M, Wicke M, Yu Y, Zheng X (2016). Tensorflow: large-scale machine learning on heterogeneous distributed systems. http://arxiv.org/abs/1603.04467.

[ref-2] Abdulmunem AA, Abutiheen ZA, Aleqabie HJ (2021). Recognition of corona virus disease (COVID-19) using deep learning network. International Journal of Electrical & Computer Engineering.

[ref-3] Ahmed E, Jones M, Marks TK (2015). An improved deep learning architecture for person re-identification.

[ref-4] Apostolopoulos ID, Aznaouridis SI, Tzani MA (2020). Extracting possibly representative COVID-19 Biomarkers from X-Ray images with deep learning approach and image data related to pulmonary diseases. Journal of Medical and Biological Engineering.

[ref-10] Bachir (2020). COVID-19 X-ray images. https://www.kaggle.com/bachrr/covid-chest-xray.

[ref-5] Calik N, Kurban OC, Yilmaz AR, Yildirim T, Ata LD (2019). Large-scale offline signature recognition via deep neural networks and feature embedding. Neurocomputing.

[ref-6] Chen H, Guo J, Wang C, Luo F, Yu X, Zhang W, Li J, Zhao D, Xu D, Gong Q, Liao J, Yang H, Hou W, Zhang Y (2020). Clinical characteristics and intrauterine vertical transmission potential of COVID-19 infection in nine pregnant women: a retrospective review of medical records. The Lancet.

[ref-8] Ciregan D, Meier U, Schmidhuber J (2012). Multi-column deep neural networks for image classification.

[ref-11] Das D, Santosh KC, Pal U (2020). Truncated inception net: COVID-19 outbreak screening using chest X-rays. Physical and Engineering Sciences in Medicine.

[ref-12] Dean J, Corrado G, Monga R, Chen K, Devin M, Mao M, Ranzato M, Senior A, Tucker P, Yang K, Le Q, Ng A (2012). Large scale distributed deep networks.

[ref-9] Faizan S (2020). COVID-19 in X-Ray Images. GitHub.

[ref-13] Frid-Adar M, Diamant I, Klang E, Amitai M, Goldberger J, Greenspan H (2018). GAN-based synthetic medical image augmentation for increased CNN performance in liver lesion classification. Neurocomputing.

[ref-14] Goodfellow IJ, Pouget-Abadie J, Mirza M, Xu B, Warde-Farley D, Ozair S, Courville A, Bengio Y (2014). Generative adversarial nets.

[ref-15] Goyal P, Dollár P, Girshick R, Noordhuis P, Wesolowski L, Kyrola A, Tulloch A, Jia Y, He K (2017). Accurate, large minibatch sgd: training imagenet in 1 hour. http://arxiv.org/abs/1706.02677.

[ref-16] Hinton G, Deng L, Yu D, Dahl G, Mohamed A-R, Jaitly N, Senior A, Vanhoucke V, Nguyen P, Sainath T, Kingsbury B (2012). Deep neural networks for acoustic modeling in speech recognition: The shared views of four research groups. IEEE Signal processing magazine.

[ref-17] Hinton GE, Salakhutdinov RR (2006). Reducing the dimensionality of data with neural networks. Science.

[ref-18] Hinton G, Srivastava N, Swersky K (2012). Neural networks for machine learning lecture 6a overview of mini-batch gradient descent. http://www.cs.toronto.edu/~hinton/coursera/lecture6/lec6.pdf.

[ref-19] Ienca M, Vayena E (2020). On the responsible use of digital data to tackle the COVID-19 pandemic. Nature Medicine.

[ref-20] Ismael AM, Şengür A (2021). Deep learning approaches for COVID-19 detection based on chest X-ray images. Expert Systems with Applications.

[ref-21] Jain P, Netrapalli P, Kakade SM, Kidambi R, Sidford A (2017). Parallelizing stochastic gradient descent for least squares regression: mini-batching, averaging, and model misspecification. Journal of Machine Learning Research.

[ref-22] Joshi A, Dey N, Santosh KC (2020). Intelligent systems and methods to combat covid-19.

[ref-23] Khan AI, Shah JL, Bhat MM (2020). Coronet: a deep neural network for detection and diagnosis of COVID-19 from chest x-ray images. Computer Methods and Programs in Biomedicine.

[ref-24] Kingma DP, Ba J (2014). Adam: a method for stochastic optimization. http://arxiv.org/abs/1412.6980.

[ref-25] Le QV, Ngiam J, Coates A, Lahiri A, Prochnow B, Ng AY (2011). On optimization methods for deep learning.

[ref-26] Lee H, Grosse R, Ranganath R, Ng AY (2011). Unsupervised learning of hierarchical representations with convolutional deep belief networks. Communications of the ACM.

[ref-27] Li L, Qin L, Xu Z, Yin Y, Wang X, Kong B, Bai J, Lu Y, Fang Z, Song Q, Cao K, Liu D, Wang G, Xu Q, Fang X, Zhang S, Xia J, Xia J (2020). Using artificial intelligence to detect COVID-19 and community-acquired pneumonia based on pulmonary CT: evaluation of the diagnostic accuracy. Radiology.

[ref-28] Lu J, Behbood V, Hao P, Zuo H, Xue S, Zhang G (2015). Transfer learning using computational intelligence: a survey. Knowledge-Based Systems.

[ref-29] Maas AL, Hannun AY, Ng AY (2013). Rectifier nonlinearities improve neural network acoustic models. Proceedings of ICML.

[ref-30] Misztal K, Agnieszka P, Martyna D-K, Michał W, Aleksandra K-M, Marcin H (2020). The importance of standardisation—COVID-19 CT & radiograph image data stock for deep learning purpose. Computers in Biology and Medicine.

[ref-7] Mooney P (2020). Chest X-Ray Images (Pneumonia). https://www.kaggle.com/paultimothymooney/chest-xray-pneumonia.

[ref-31] Oh Y, Park S, Ye JC (2020). Deep learning covid-19 features on cxr using limited training data sets. IEEE Transactions on Medical Imaging.

[ref-32] Ozturk T, Talo M, Yildirim EA, Baloglu UB, Yildirim O, Acharya UR (2020). Automated detection of COVID-19 cases using deep neural networks with X-ray images. Computers in Biology and Medicine.

[ref-33] Pereira RM, Bertolini D, Teixeira LO, Silla CN, Costa YM (2020). COVID-19 identification in chest X-ray images on flat and hierarchical classification scenarios. Computer Methods and Programs in Biomedicine.

[ref-34] Perumal V, Narayanan V, Rajasekar SJS (2020). Detection of COVID-19 using CXR and CT images using transfer learning and Haralick features. Applied Intelligence.

[ref-35] Razzak MI, Naz S, Zaib A (2018). Deep learning for medical image processing: overview, challenges and the future. Classification in BioApps.

[ref-36] Ruder S (2016). An overview of gradient descent optimization algorithms. http://arxiv.org/abs/1609.04747.

[ref-37] Sahlol AT, Yousri D, Ewees AA, Al-Qaness MAA, Damasevicius R, Elaziz MA (2020). COVID-19 image classification using deep features and fractional-order marine predators algorithm. Scientific Reports.

[ref-38] Salehinejad H, Valaee S, Dowdell T, Colak E, Barfett J (2018). Generalization of deep neural networks for chest pathology classification in x-rays using generative adversarial networks.

[ref-39] Samangouei P, Kabkab M, Chellappa R (2018). Defense-gan: protecting classifiers against adversarial attacks using generative models. http://arxiv.org/abs/1805.06605.

[ref-40] Santosh KC (2020a). COVID-19 prediction models and unexploited data. Journal of Medical Systems.

[ref-41] Santosh KC (2020b). AI-driven tools for coronavirus outbreak: need of active learning and cross-population train/test models on multitudinal/multimodal data. Journal of Medical Systems.

[ref-42] Santosh KC (2021). COVID-19: prediction, decision-making, and its impacts.

[ref-43] Shams M, Elzeki O, Abd Elfattah M, Hassanien A (2020a). http://dx.doi.org/10.17632/fvk7h5dg2p.3.

[ref-44] Shams MY, Elzeki OM, Abd Elfattah M, Medhat T, Ella Hassanien A (2020b). Why are generative adversarial networks vital for deep neural networks? A case study on COVID-19 chest X-ray images. Big data analytics and artificial intelligence against COVID-19: innovation vision and approach.

[ref-45] Shi H, Han X, Jiang N, Cao Y, Alwalid O, Gu J, Fan Y, Zheng C (2020). Radiological findings from 81 patients with COVID-19 pneumonia in Wuhan, China: a descriptive study. Lancet Infectious Diseases.

[ref-46] Shorten C, Khoshgoftaar TM (2019). A survey on image data augmentation for deep learning. Journal of Big Data.

[ref-47] Sohrabi C, Alsafi Z, O'Neill N, Khan M, Kerwan A, Al-Jabir A, Iosifidis C, Agha R (2020). World Health Organization declares global emergency: a review of the 2019 novel coronavirus (COVID-19). International Journal of Surgery.

[ref-48] Struyf T, Deeks JJ, Dinnes J, Takwoingi Y, Davenport C, Leeflang MMG, Spijker R, Hooft L, Emperador D, Dittrich S, Domen J, Horn SRA, Van den Bruel A (2020). Signs and symptoms to determine if a patient presenting in primary care or hospital outpatient settings has COVID-19 disease. Cochrane Database of Systematic Reviews.

[ref-49] Ting DSW, Carin L, Dzau V, Wong TY (2020). Digital technology and COVID-19. Nature Medicine.

[ref-50] Toğaçar M, Ergen B, Cömert Z (2020). COVID-19 detection using deep learning models to exploit social mimic optimization and structured chest X-ray images using fuzzy color and stacking approaches. Computers in Biology and Medicine.

[ref-51] Tran PT (2019). On the convergence proof of amsgrad and a new version. IEEE Access.

[ref-52] Ucar F, Korkmaz D (2020). COVIDiagnosis-net: deep bayes-squeezeNet based diagnostic of the coronavirus disease 2019 (COVID-19) from X-ray images. Medical Hypotheses.

[ref-53] Waheed A, Goyal M, Gupta D, Khanna A, Al-Turjman F, Pinheiro PR (2020). Covidgan: data augmentation using auxiliary classifier gan for improved covid-19 detection. IEEE Access.

[ref-54] Wang CJ, Ng CY, Brook RH (2020). Response to COVID-19 in Taiwan: big data analytics, new technology, and proactive testing. JAMA.

[ref-55] Wang Z, Wang J, Wang Y (2018). An intelligent diagnosis scheme based on generative adversarial learning deep neural networks and its application to planetary gearbox fault pattern recognition. Neurocomputing.

[ref-56] Wang W, Xu Y, Gao R, Lu R, Han K, Wu G, Tan W (2020). Detection of SARS-CoV-2 in different types of clinical specimens. JAMA.

[ref-57] World Health Organization (2020). WHO director-general’s remarks at the media briefing on 2019-nCoV on 11 February 2020. https://www.who.int/director-general/speeches/detail/who-director-general-s-remarks-at-the-media-briefing-on-2019-ncov-on-11-february-2020.

[ref-58] Xu Y, Mo T, Feng Q, Zhong P, Lai M, Eric I, Chang C (2014). Deep learning of feature representation with multiple instance learning for medical image analysis.

[ref-59] Yosinski J, Clune J, Bengio Y, Lipson H (2014). How transferable are features in deep neural networks?.

